# Full-Scale Pore Structure Characteristics and the Main Controlling Factors of Mesoproterozoic Xiamaling Shale in Zhangjiakou, Hebei, China

**DOI:** 10.3390/nano11020527

**Published:** 2021-02-18

**Authors:** Liangwei Xu, Keji Yang, Hao Wei, Luofu Liu, Xiao Li, Lei Chen, Tong Xu, Ximeng Wang

**Affiliations:** 1 School of Earth and Space Sciences, Peking University, Beijing 100871, China; xlwcumtbcupb@163.com; 2 Hebei Key Laboratory of Strategic Critical Mineral Resources, Hebei GEO University, Shijiazhuang 050031, China; 3 State Key Laboratory of Petroleum Resources and Prospecting, China University of Petroleum, Changping, Beijing 102249, China; liuluofucupb@126.com (L.L.); chenlei19880804@163.com (L.C.); xutong1995@yeah.net (T.X.); wxm123098@sina.com (X.W.); 4 Experimental Practice Teaching Centre of Hebei GEO University, Shijiazhuang 050031, China; ronghaiwei@163.com (H.W.); xiao223623@126.com (X.L.)

**Keywords:** Ximaling shale, full-scale pore structure, organic pores, pore size distribution, residual hydrocarbon

## Abstract

Nanoscale pore structure characteristics and their main controlling factors are key elements affecting the gas storage capacity, permeability, and the accumulation mechanism of shale. A multidisciplinary analytical program was applied to quantify the pore structure of all sizes of Xiamaling shale from Zhangjiakou, Hebei. The result implies that Mercury injection porosimetry (MIP) and low-pressure N_2_ curves of the samples can be divided into three and four types, respectively, reflecting different connectivity performances. The maximum CO_2_ adsorbing capacity increases with increasing total organic carbon (TOC) content, pore volume (PV), and surface area (SA) of the micropores are distributed in a three-peak type. The full-scale pore structure distribution characteristics reveal the coexistence of multiple peaks with multiple dominant scales and bi-peak forms with mesopores and micropores. The porosity positively correlates with the TOC and quartz content, but negatively correlates with clay mineral content. Organic matter (OM) is the main contributor to micropore and mesopore development. Smectite and illite/smectite (I/S) assist the development of the PV and SA of pores with different size. Illite promotes the development of the nanoscale PV, but is detrimental to the development of the SA. Thermal maturity controls the evolution of pores with different size, and the evolution model for the TOC-normalized PVs of different diameter scales is established. Residual hydrocarbon is mainly accumulated in micropores sized 0.3 to 1.0 nm and mesopores sized 40 nm, 2 nm and less than 10 nm. Since the samples were extracted, the pore space occupied by residual hydrocarbon was released, resulting in a remarkable increase in PV and SA.

## 1. Introduction

Shale gas is mainly adsorbed on the surface of mineral particles or organic matter (OM), or is stored in a free state in nanoscale pores or a dissolved state in kerogen and bitumen [1,2,3,4]. Nanoscale pores not only provide storage space for shale gas but also provide a geological basis for later reservoir exploitation. Therefore, nanoscale pore structure is an important element in the evaluation of the gas storage capacity and seepage performance of shale gas and a key indicator of whether shale gas has commercial exploitation value [1,5,6].

However, owing to the complexity and variability in shale pores, conventional reservoir porosity testing methods are far from sufficient for the requirements of characterization. To better characterize the pore structure of shale, a variety of technical means should be combined. Accordingly, a range of qualitative and quantitative methods have previously been applied by scholars. The qualitative methods include computerized tomography (CT) scanning, transmission electron microscopy (TEM), field emission scanning electron microscopy (FE-SEM), broad-ion beam SEM (BIB-SEM), and focused ion beam polishing in combination with SEM (FIB-SEM) [7,8,9,10,11]. These qualitative image observation technologies have previously been widely applied to observe pore sizes and distributions and the geometry and connectivity of pore networks. Image-based visible nanoscale pore information can be extracted via point counting in Image J software (V1.8.0.112, 22 July 2019) [12,13]. Quantitative methods, such as small angle and ultra-small angle neutron scattering, gas adsorption (CH_4_, CO_2_, N_2_), and mercury intrusion porosimetry (MIP), have been used to measure pore structure parameters at nm-scale resolution. To quantify pore structures of all sizes, combinations of MIP, CO_2_, and N_2_ adsorption have been successfully used by numerous scholars [2,6,14,15,16].

Under actual geological conditions, the nanoscale pore structure of shale is constantly changing due to the combined effect of diagenesis and the thermal transformation of OM and kerogen [17,18]. OM and kerogen are the material bases for the hydrocarbon generation of shale, and play a decisive role in the generation and development of organic pores [19]. According to Jiarvie et al. [20], the porosity of shale increases by 4.9% with organic carbon consumption of 35%. According to the simulation results of Modica and Lapierre [21], each gram of organic carbon generally contributes 0.8–0.85% to the pores of shale at the end of the gas generation of OM. Therefore, convertible OM results in pyrolysis and leads to the generation and discharge of hydrogen [22] at a certain thermal maturity. As large numbers of OM pores are generated, the residual hydrogen in the shale inevitably influences the pore system. The inorganic minerals in shale mainly include brittle minerals such as quartz and feldspar, whereas clay minerals are dominated by smectite, illite/smectite (I/S), and illite. These minerals determine the formation and evolution of a framework of mineral pores in shale during the process of diagenesis [1,22]. However, current research on factors affecting the full-scale pore structure primarily involves postmature or extremely mature marine gas generation shales, with a thermal maturity greater than 2.0% [6,23,24,25], whereas research on the main factors controlling the full-scale pore structure of low- and middle-mature marine shale with favourable aerogenesis is scarce. High- and over-mature shale is the product of low- and middle-mature shale during diagenesis and hydrocarbon generation, and immature shale with different pore structure characteristics inevitably generates high- and over-mature shale with different accumulation effects. In particular, low- and middle-mature shale formations with strong hydrocarbon generation ability contain large amounts of residual hydrocarbon, which leads to nonnegligible influences on the formation and development of the pore structure and subsequently changes the gas storage and filtration ability of shale, influencing exploitation and gas generation.

Therefore, based on the aforementioned status, a combination of MIP, low-pressure N_2_ and CO_2_ adsorption was applied (1) to characterize the complex full-scale pore size distribution of Xiamaling shale originating from Zhangjiakou, Hebei, China; (2) to reveal the relationship between the pore structure and affecting factors; and (3) to provide evidence for evaluating the reservoir performance of Xiamaling shale and the shale of different date and formation of the world. The MIP and N_2_ low pressure adsorption curve classification of this research can be used to evaluate the development degree, morphological characteristics, and connectivity of pores with different pore sizes in shale, and to show the contribution degree of pores with different pore sizes to PV and SA. This study provides important evidence for understanding and evaluating the desorption, diffusion, seepage, migration, and later exploitation of shale gas in different occurrence states.

## 2. Geological Setting

**Figure 1:**
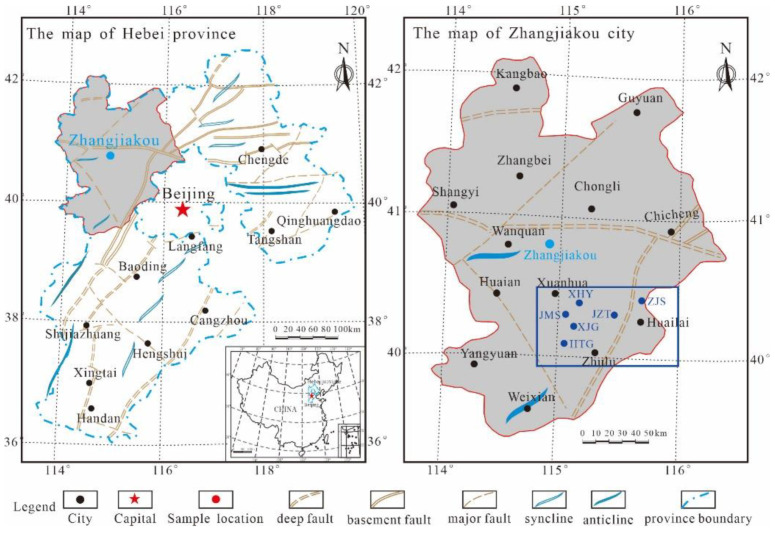
Structural outline of the research area and the sampling point locations.

The research area is located in Zhangjiakou, Hebei (Figure 1). Xiamaling shale is widely distributed in the villages of Xiahuayuan, Zhaojiashan, Jingjitun, and Xiajiagou. The Zhaojiashan and Xiajiagou areas are the deposition centre of Xiamaling Formation, with a maximum cumulative thickness of 587 m and thinning from west to east, and the section exposure is almost complete, in which a standard section has developed. The Xiamaling Formation unconformably contacts the overlying Changlongshan Formation and underlying Tieling Formation and can be divided into four sections [26,27]. The thick of the third section is up to 350 m and contains black carbonaceous, silty and grey-black siliceous shale, as well as oil shale accumulated by red algae and brown algae, indicating that the Xiamaling shale in this region has superior hydrocarbon generation potential, with favourable shale gas exploration prospects. Additionally, according to the latest isotopic timing results, the Ximaling shale formation was deposited 1368 Ma years ago, and formed at Jixian system in Precambrian, indicating that the Ximaling shale is the oldest marine shale found in China, and it is one of the oldest ancient marine shales in the world [28,29].

## 3. Samples and Experiments

In total, 18 fresh outcrop shale samples from the six villages of Zhaojiashan, Jizhentun, Xiajiagou, Huangtugang, Jimingshan, and Xiahuayuan were collected for the precursory studies. The six sections all include organic-rich Xiamaling marine shale, and the locations are marked in Figure 1. The experimental samples were mainly black and carbonaceous shale. During the sample collection process, fresh samples were collected 2–3 m below the surface. As the experimental samples were collected from the outcrops of the Xiamaling shale in Zhangjiakou, any weathered surface material on the collected samples was removed, and the samples were thoroughly cleaned using ethanol to remove possible surface contamination. Each of the selected samples was split into several aliquots for analysis of the mineralogical composition, pore morphology, and pore size distributions, for organic petrographic analysis, and the extraction of residual hydrocarbon.

### 3.1. Basic Geochemistry and Mineralogy

The TOC content was measured by an LEC CS-230 carbon-sulphur analyser after treatment with HCl to remove carbonates according to the Chinese National Standard GB/T18602-2001 and GB/T19145-2003. Because no “true” vitrinite was found in the shale, vitrinite-like maceral reflectance was used to characterize the thermal maturity. The measurements were conducted on polished blocks under reflected light using a 3Y microphotometric system equipped with an oil-immersion objective lens and photometer. The vitrinite-like maceral reflectance was then converted to the equivalent vitrinite reflectance (EqRo) using the equation proposed by Xiao et al. [30].

X-ray diffraction (XRD) was performed using a Bruker D8 Advance X-ray diffractometer with a scan range of 3° to 85° (2θ) and a scan speed of 4°/min. The analysis was conducted on powdered bulk shales and clay fractions to determine the mineralogical composition and identify the clay minerals, respectively. The area under the curve for the major peaks of each mineral was used to estimate the relative mineral percentages of the shale with correction for Lorentz polarization [31].

### 3.2. Mercury Injection Porosimetry

Porosity and macropore characterization was performed on cubic samples sized approximately 1 × 1 × 1 cm^3^ using an AutoPore IV 9510 Micrometerics instrument (Micromeritics Instrument, Norcross, GA, USA) at 19 °C and a humidity of 18%. The bulk and skeletal densities used for determining the porosity of the sample could be obtained from the mercury intrusion measurements performed at 0 and 413 MPa, respectively. The measured spans of the pore diameter were approximately 3 nm–1000 μm, the mercury contact angle was 130°, and the surface tension was 485 dyne/cm. The accuracy of the mercury injection volume was 0.1 mL, and the macropore size distribution was calculated by means of the Washburn formula [32]. Each sample prepared for the MIP test was oven dried for 48 h at 110 °C to remove adsorbed moisture and volatile matter and then cooled to room temperature before measurements were obtained.

### 3.3. Residual Hydrocarbon Extraction

Prior to the Soxhlet extraction experiments, free water was removed from the shale samples for 24 h at 48 °C. Then, the samples were crushed and sieved by 80–120 meshes. Approximately 40 g of the measured sample grains was used for Soxhlet extraction for 72 h with a 25:2 vol/vol mixture of dicholoromethane and methanol. After the extracted liquid mixture was concentrated using a rotary evaporator, the mixture was transferred and weighed in a 4 cm^3^ glass vial, and the weight of the concentrated liquid mixture represented the content of residual hydrocarbon. The original samples and their corresponding extracted solid residues were placed in a vacuum oven and held for 24 h at 90 °C. Then, low-pressure N_2_ and CO_2_ adsorption measurements were obtained.

### 3.4. Low-Pressure N_2_ and CO_2_ Adsorption

Low-pressure N_2_ and CO_2_ adsorption were performed using a Quantachrome Autosorb-1 apparatus SA analyser at the State Key Laboratory of Heavy Oil Processing in China University of Petroleum, Beijing. The 2–3 g shale sample was crushed into grains of approximately 60–100 mesh (250–150 μm) to obtain nanoscale pore structure parameters. Prior to the low-pressure N_2_ and CO_2_ adsorption measurements, the selected samples were automatically degassed under vacuum for approximately 12 h at 110 °C to remove air, free water, and other gases. The conditions of N_2_ and CO_2_ adsorption measurements were maintained at 77.35 K at 101.3 kPa and 273.15 K, respectively. The equilibrium times of the measurements, over which the pressure had to be stable, were short. Equilibrium times of 30 s and 45 s were set for N_2_ and CO_2_, respectively. The N_2_ and CO_2_ adsorption experiment relative pressures ranged from 0.009 to 0.995 and 0.0001 to 0.032, respectively. A density functional theory (DFT) molecular model was used to determine the pore size distributions (PSDs) of the micropores and mesopores since this technique can provide a more accurate approach for pore size analysis. The N_2_ adsorption data were interpreted by applying Langmuir analyses and the multi-point Barrett-Emmett-Teller (BET) model for the SA and the Barrett-Joyner-Halenda (BJH) model for the PV of the mesopores. The SA and PV characteristics of micropores measured by CO_2_ adsorption were analysed using the BET, Langmuir, and DFT models, and detailed descriptions of these theories were previously reported in various studies [9,33,34].

### 3.5. Determination of Total Porosity

The porosity of the shale samples was calculated as per the differences between bulk density (ρ_bulk_) and skeletal density (ρ_skeletal_); detailed descriptions of these experimental procedures have already been reported in various literature works [9,15,23,24,33]. ρ_bulk_ of the cylindrical plug samples were determined by caliper measurements. The drilled plug samples with diameter 2.54 cm and height 4 cm were dried at 110 °C overnight to remove free water. The samples were then weighed in air before and after coated by paraffin of known density, then the paraffin-coated samples were weighed both in air and water of known density to obtain the samples’ bulk volume, finally the ρ_bulk_ was calculated by weight in air and bulk volume. After the analysis, the paraffin was crushed between 20 and 40 mesh sizes (380–830 μm) and dried at 110 °C in a vacuum for 24 h. ρ_skeletal_ was measured with a helium pycnometer at pressure less than 17.4 psi according to Boyle’s Law. Then the total porosity (φ) of the Xiamaling shales were calculated by the differences between the ρ_skeletal_ and ρ_bulk_, according to the following equation:

(1) $$\varphi \;=\;(1\;-\;\frac{\rho_{\mathrm{bulk}}}{\rho_{\mathrm{skeletal}}})\;\times \;100\%$$

## 4. Results

### 4.1. Basic Organic Geochemistry and Mineralogy

The TOC content of the Xiamaling shale varies from 0.55% to 7.60%, with an average value of 3.10%, and the equivalent vitrinite reflectance ranges between 0.52% and 1.88%, indicating that the shale is a “good” source rock and is in the low- and high-maturation stages. Six samples were extracted by dicholoromethane and methanol, and the extracted OM content ranges between 0.79% and 1.93%, with an average value of 1.16% (Table 1).

The Xiamaling shale samples are dominated by clay minerals (21.5–53.8%, average 40.57%) and quartz (21.8–48.4%, average 34.26%), followed by calcite (5.2–32.4%, average 16.59%) and feldspar (1.7–11.4%, average 5.76%). A low content of pyrite (0–7.2%, average 2.82%) is also present. The clay minerals mainly consist of I/S (8.5–40.0%, average 19.86%), followed by illite (3.8–27.4%, average 9.75%) and smectite (3.6–17.9%, average 8.84%), and the content of chlorite is relatively low, with average values less than 3.0% (Table 1).

### 4.2. Pore Characterization Based on MIP

The macro-PV of the Xiamaling shale samples ranges from 0.0016 cm^3^/g, as noted in sample JMS-4, to 0.0136, as noted sample XHY-4. The macropore SA ranges from 0.21 m^2^/g, as noted in sample JZT-6, to 2.67 m^2^/g, as noted in sample JZT-1. The porosity of the Xiamaling shale samples is in the range of 0.57–3.22% (Table 2).

**Figure 2:**
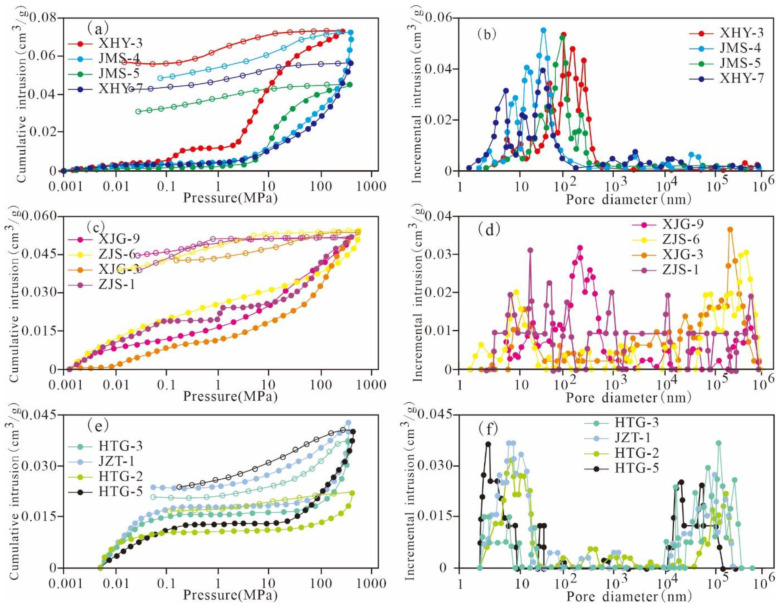
Mercury intrusion curve characteristics and pore type classification of Mesoproterozoic Xiamaling shale samples in Zhangjiakou, Hebei.4.3. Mesopore and micropore characterization based on gas adsorption. (**a**,**b**): the first type of MIP curve and its corresponding pores; (**c**,**d**): the second type of MIP curve and its corresponding pores; (**e**,**f**): the third type of MIP curve and its corresponding pores).

The different hysteresis loops of MIP curves reflect different pore characteristics and serve as the basis for revealing the pore characteristics in the samples [35,36]. The MIP curves and reflected pores of the Xiamaling shale samples can be divided into three types according to the morphology of the hysteresis loops (Figure 2).

The first type is represented by XHY-3, JMS-4, JMS-5, and XHY-7 and is predominantly composed of pores with widths less than 100 nm, which implies that the mesopores and macropores are more developed. The mercury-in curve increases sharply at approximately 5 MPa, and the mercury-out curve exhibits a convex or a horizontal shape. The hysteresis loop of the MIP curve is wide, with a large difference between the mercury-in volume and the mercury-out volume. This finding implies that most of the pores are open, with favourable pore connectivity. There are large numbers of connected pores with small pore widths; however, macropores do not develop, which is beneficial to the desorption, diffusion, and transfusion of shale gas. The second type is represented by XJG-9, ZJS-6, XJG-3, and ZJS-1 and is characterized by full-scale pores. The hysteresis loop of the MIP curve is wide, with a certain amount of mercury-out as well as a large difference between the mercury-in volume and mercury-out volume. This characteristic indicates that most of the pores are open, with favourable connectivity. Both the macropores and mesopores are developed in the shale, which is also beneficial to the desorption, diffusion, and transfusion of shale gas. The third type includes HTG-3, JZT-1, HTG-2, and HTG-5 and is characterized by pores with diameters greater than 10,000 nm and less than 10 nm. During the initial stage, the mercury-in pressure is low, and the mercury-in speed is relative high. There is a flat stage in the range of 0.1–10 MPa, and the mercury-in amount and the mercury-out speed increase after 10 MPa. The hysteresis loop of the MIP curve is narrow, with a certain mercury-out volume and a small difference between the mercury-in volume and mercury-out volume. This characteristic indicates that there are relatively small numbers of open pores and large numbers of disconnected pores, which are caused by undeveloped pores with diameters of 10–10,000 nm. This type of pore is detrimental to the desorption, diffusion, and transfusion of shale gas.

The main pore structure parameters measured by the low-pressure gas adsorption of the Xiamaling shale are summarized in Table 3. The PV of micropores and mesopores are in the range of 0.0021–0.0245 cm^3^/g and 0.0046–0.0222 cm^3^/g, respectively. The SAs of micropores and mesopores are in the ranges of 7.3622–41.2702 m^2^/g and 4.2128–19.3278 m^2^/g, respectively.

**Figure 3:**
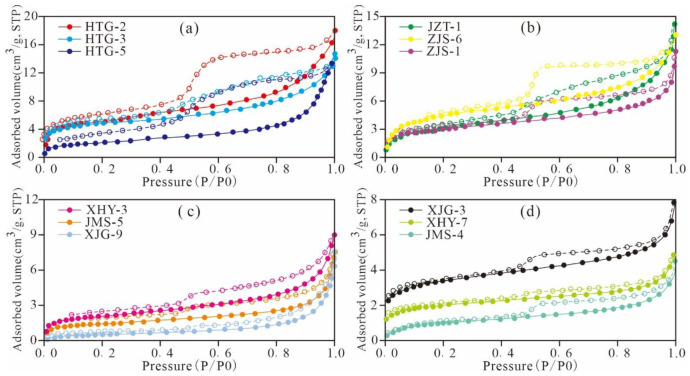
N_2_ adsorption and desorption isotherms of representative samples of the Mesoproterozoic Xiamaling shale in Zhangjiakou, Hebei ((**a**), the first type of N_2_ adsorption curve; (**b**), the second type of N_2_ adsorption curve; (**c**), the third type of N_2_ adsorption curve; (**d**), the fourth type of N_2_ adsorption curve).

In this study, the morphological characteristics from the low-pressure N_2_ adsorption curves of shale samples from the Xiamaling Formation were statically analysed. According to the IUPAC classification, the samples were generally classified into four types (Figure 3). The first type is represented by HTG-2, HTG-3, and HTG-5. The curves exhibit a certain amount of N_2_ adsorption and a wide hysteresis loop, which corresponds to type H2 isotherms as defined by IUPAC [37]. The second type is represented by JZT-1, ZJS-6, and ZJS-1. The curve shape is similar to that of the first type of curve, but the curves exhibit a large adsorption capacity at P/P_0_ of approximately 0.995, demonstrating an unsaturated adsorption state and a large hysteresis loop, which is similar to type H2 but has the characteristics of type H3 as defined by IUPAC [37]. The results indicate that these samples have larger pores than the first type, including wedge or V-type pores with both ends open. The third type is represented by XHY-3, JMS-5, and XJG-9. These curves exhibit a certain adsorption content at the low-pressure and high-pressure stage and are steeper at saturated vapour pressure. The hysteresis is narrower than those of the aforementioned two types and close to that of type H3, as defined by IUPAC [37]. The results show that the pores are mainly flat slit structures, cracks, and wedge structures, and the hysteresis loop is mainly related to clay and other flaky granular materials. The fourth type is represented by XJG-3, XHY-7, and JMS-4. These curves are less to the third type of curve, except the increment at the saturated vapor pressure is smaller than that of the third type. These curves are similar to the H3-type curve defined by IUPAC but exhibit the characteristics of the H4-type curve [37] The results show that the pores are slit-shaped pores and wedge-shaped semi-closed pores with parallel walls, and a certain amount of ink bottle-shaped pores are noted.

**Figure 4:**
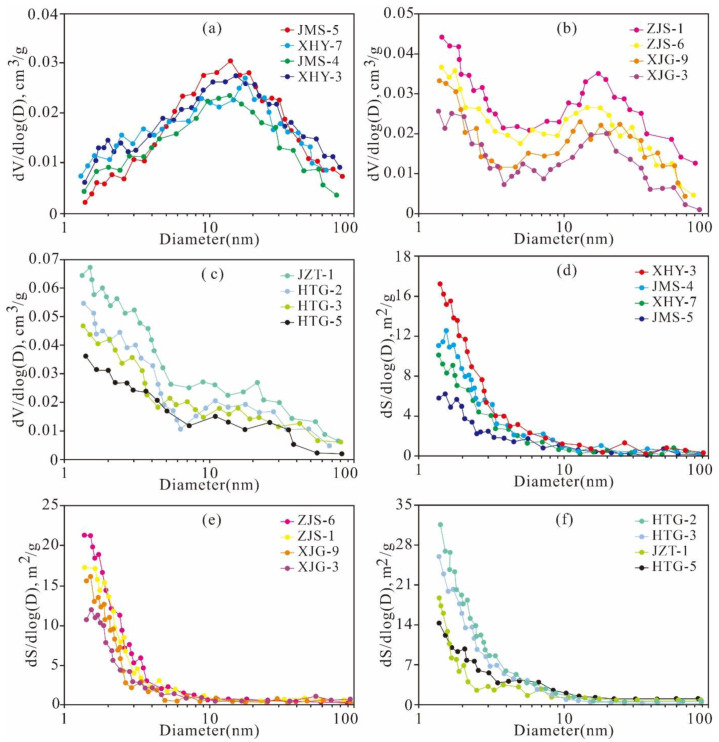
PV (**a**–**c**) and SA (**d**–**f**) distribution with pore size derived from the N_2_ adsorption branch for the isotherms of representative samples of Mesoproterozoic Xiamaling shale in Zhangjiakou, Hebei.

The PSDs of the Xiamaling shales calculated by the BJH model are presented in Figure 4. The plot of dV/d(logD) versus D clearly reveals that the PV is mainly contributed by the mesopores and micropores and can be divided into three types. The first type is represented by JMS-5, XHY-7, JMS-4, and XHY-3. The TOC of the samples is less than 1.0%, and the clay mineral content is high. The PV distribution is post-peak type, indicating that fewer micropores were generated and that the mesopores were more developed. This finding is attributed to the fact that the samples with low TOC content did not contain the numerous micropores generated by hydrocarbon generation of OM. Clay minerals provide a small number of micropores and a large number of mesopores. The second type is represented by ZJS-1, ZJS-6, XJG-3, and XJG-9. The TOC content varies from 1.0% to 3.0%, and there is a low content of clay minerals. The PV exhibits a bimodal distribution. The main maximum value is distributed in the range of 1–10 nm diameter, and the other is distributed at 2–10 nm diameter. The results showed that the micropores and narrow mesopores in these samples were more developed and contributed the most to the PV. The third type is represented by JZT-1, HTG-2, HTG-3, and HTG-5. The TOC content is greater than 3.0%, and the clay mineral content is much lower than that of the second type. The PV exhibits a pre-peak distribution, indicating that the micropores of these samples are more developed and constitute most of the PV.

The log differential SAs of all the studied samples exhibit broad porosity ranges and approximately similar variation tendencies of bimodal distributions with modes of approximately 1.5 nm and 2–3 nm (Figure 4d–f). The peak values of the log differential SA obviously increase with the TOC content. The pores with diameters <10 nm contribute relatively significantly to the total SA.

**Figure 5:**
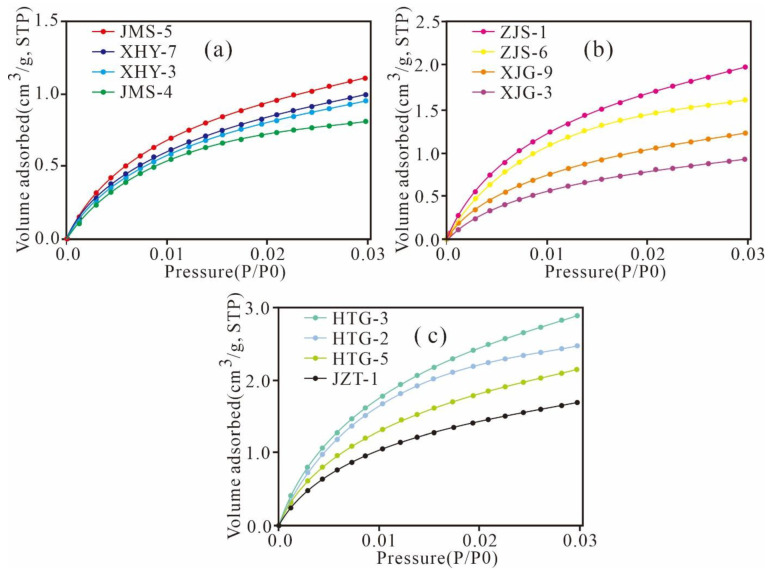
CO_2_ adsorption isotherms of the Mesoproterozoic Xiamaling shale in Zhangjiakou, Hebei ((**a**), TOC < 1.0%; (**b**), 1.0% < TOC < 3.0%; (**c**), TOC > 3.0%).

The CO_2_ adsorption isotherms of the Xiamaling shale exhibit similar trends and variations (Figure 5), which manifest as type I according to the IUPAC and are characterized by microporous solids [37]. Obviously as TOC content increases, the maximum volume of the CO_2_ adsorption content increased from 0.83 to 2.96 cm^3^/g, indicating that the number of micropores increased with increasing TOC content.

**Figure 6:**
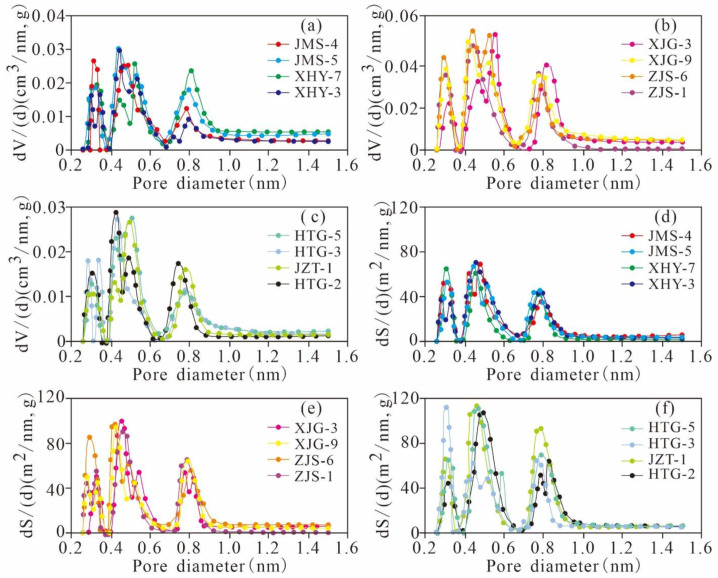
Micropore PV (**a**–**c**) and SA (**d**–**f**) distribution derived from the CO_2_ adsorption isotherms of the Mesoproterozoic Xiamaling shale in Zhangjiakou, Hebei.

The micropore size distribution of the Xiamaling shale obtained by the DFT model exhibits a high log differential PV and SA and similar distribution characteristics (Figure 6). There are three stable peaks at approximately 0.3~0.4 nm, 0.45–0.6 nm and 0.7~1.0 nm, which exhibit the same increasing and decreasing tendencies. The peak values of PV and SA generally increase with TOC content, which indicates a high abundance of micropore distributions of the corresponding pore size, and the abundance of micropores increases with increasing TOC content. Furthermore, micropores sized 0.45–0.7 nm are the most prevalent in the distribution, and pores with diameters of approximately 0.3~0.45 nm and 0.7~1.0 nm also significantly contribute to the PV and SA, respectively.

## 5. Discussion

### 5.1. Relationship between TOC and Quartz and Clay Minerals

**Figure 7:**
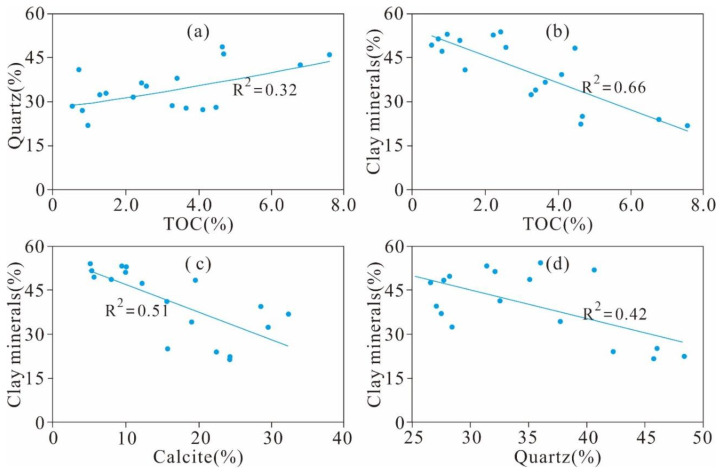
Cross plots of (**a**) quartz vs. TOC; (**b**) clays vs. TOC; (**c**) clay vs. calcite; (**d**) clay vs. quartz.

The TOC content of 18 shale samples exhibits a weak correlation with the quartz content (R^2^ = 0.32, Figure 7a), which is also observed in Longmaxi shale from the Sichuan Basin in South China and Devonian shale samples from the Horn River Basin [23,38]. The positive relationship indicates that the quartz is of biogenic origin. Additionally, the TOC content exhibits a negative correlation with clay minerals (R^2^ = 0.66, Figure 7b). Pan et al. [39] reported that a negative correlation is observed for samples with TOC content < 12.0%, whereas a positive correlation exists for the samples with TOC content > 12.0%. Accordingly, more samples with higher TOC contents are needed to completely estimate the relationship between the TOC content and clay minerals in the Xiamaling shales. In addition, the clay minerals of the Xiamaling shale exhibited marked negative relationships with calcite content (R^2^ = 0.51, Figure 7c) and quartz content (R^2^ = 0.42, Figure 7d).

### 5.2. Full-Scale Pore Structure Characteristics

**Figure 8:**
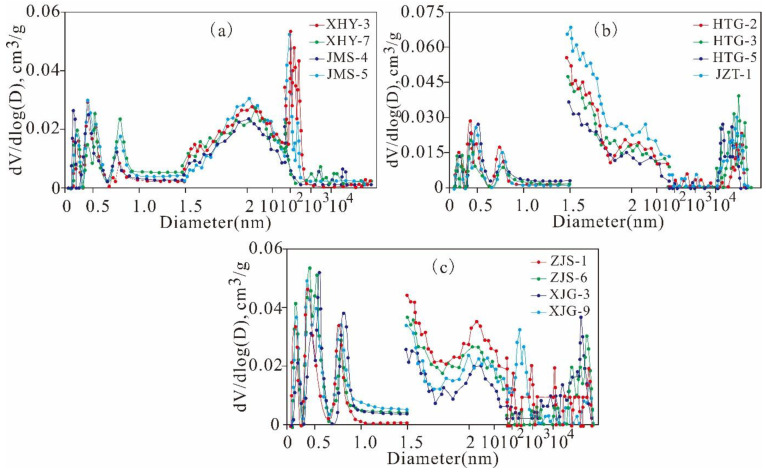
Full-scale PV distribution obtained by MIP, low-pressure N_2_ and CO_2_ adsorption of the Mesoproterozoic Xiamaling shale in Zhangjiakou, Hebei ((**a**), multimodal with multiple scale pores; (**b**), bimodal with primarily mesopores; (**c**), bimodal with primarily micropores).

As reported in previous studies, pore throats are the same as pore bodies, and the PSD curves of MIP and low-pressure N_2_/CO_2_ adsorption exhibit good correlations [6,14,24,40]. Therefore, we connect the incremental PV curves and neglect the intrinsic characteristics of the MIP and low-pressure N_2_/CO_2_ adsorption techniques. According to the combined representation results, the pore diameters of the shale mainly include the following three types:

Type I: multimodal with multiple scales of pore types, which are represented by XHY-3, XHY-7, JMS-4, and JMS-5 (Figure 8a). This type includes pores with a multi-peak diameter distribution, in addition to micropores and mesopores, and macropores contribute a significant proportion. Micropores, mesopores, and macropores are developed in these types of pores at equivalent scales, which is beneficial to shale gas reservation, migration, and exploitation. Type II: bimodal with primarily mesopores, which are represented by HTG-2, HTG-3, HTG-5, and JMS-1 (Figure 8b). This type includes pores with bimodal distributions of micropores and mesopores, which can provide large amounts of reservation space for shale gas in adsorbed and free states. Type III: bimodal with primarily micropores, which are represented by ZJS-1, ZJS-6, XJG-3, and XJG-9 (Figure 8c). This type includes pores with bimodal distributions of mesopores and micropores, which can provide large amounts of SA and are extremely beneficial for reserving shale gas in the adsorbed state.

In addition, it is worth noting that the pore classification reflected by the MIP curve and the low-pressure gas adsorption and desorption curve in this study is also applicable to shale reservoirs of different ages and strata in other regions, and the full-scale pore classification is also generally and comprehensively representative and applicable. Compared with the previous classification, these classification and pore diameter distribution features can be used to evaluate the development degree, morphological characteristics, and connectivity of pores with different pore sizes in shale more comprehensively, intuitively, and deeply, and reveal their influence on shale gas desorption, diffusion, and seepage more clearly [14,24,35,40]. Furthermore, these classifications can clearly demonstrate the contribution of pores with different sizes to PV and SA, and distinguish their influences on the occurrence, migration, and later exploitation of shale gas with different occurrence states.

### 5.3. Control Effects on Pore Structure

#### 5.3.1. Porosity and Controlling Factors

**Figure 9:**
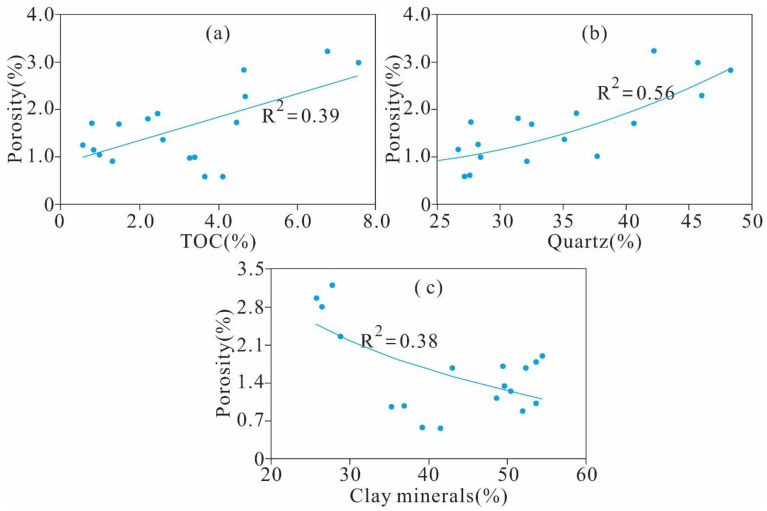
Relationships between the porosity and TOC content (**a**), quartz (**b**) and clay (**c**).

Figure 9a demonstrates that the total porosity is weakly positively correlated with the TOC content, indicating that OM plays a significant positive role in the total porosity but is not the most essential controlling factor. The positive relationship between the total porosity and TOC content was previously mentioned by many scholars [23,24,41], and some studies have reported that the total porosity is positively correlated with the TOC content but is negatively correlated with increasing TOC content for samples with TOC content higher than a certain value owing to diagenesis and compaction [19,39].

As shown in Figure 9b, the porosity is positively correlated with quartz. The biogenic quartz in marine shale may contain a large number of intraparticle pores [1,2], but this is unlikely to be the main reason for the elevated porosity of the Xiamaling shale since the TOC content increases with increasing quartz content (Figure 7a). The relationship between the total porosity and quartz content actually reflects the correlation of the porosity with the TOC content. The total porosity is negatively correlated with clay content according to the illustration of Figure 9c, indicating that the contribution of clay minerals to the total porosity of the Ximaling shale could be negligible, even though clay minerals contribute to the porosity of the shale [2,31]. The interpretation of these relationships needs to be confirmed with more detailed research, given that this is a preliminary result.

#### 5.3.2. Effect of TOC Content on Pore Structure

**Figure 10:**
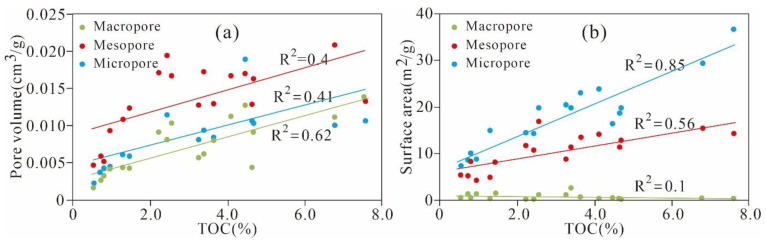
Relationship between the TOC content and pore structure in Ximaling shale ((**a**): pore volume; (**b**) surface area).

This research investigated the relationship between the TOC content and nanoscale pore structure parameters in Xiamaling shale (Figure 10). A positive linear correlation is observed between the TOC content and PV of the micropores, mesopores, and macropores (R^2^ = 0.41, 0.40 and 0.62, respectively) (Figure 10a). Strong positive relationships are also found between the TOC contents and micropore and mesopore SAs (R^2^ = 0.85, 0.56, respectively). However, the macropore SA remains stable with variation of the TOC content. The aforementioned appearance suggests that OM is the most important contributor to micro- and mesopores and that OM can be used as an important parameter to evaluate the micropores and mesopores in Xiamaling shale. The shale with higher TOC content tends to have more micropores and mesopores with larger PV and SA. The thermal maturation and hydrocarbon expulsion of OM results in the generation and development of abundant OM pores, which are mainly micro- and mesopores.

#### 5.3.3. Effect of Mineral Composition on Pore Structure

**Figure 11:**
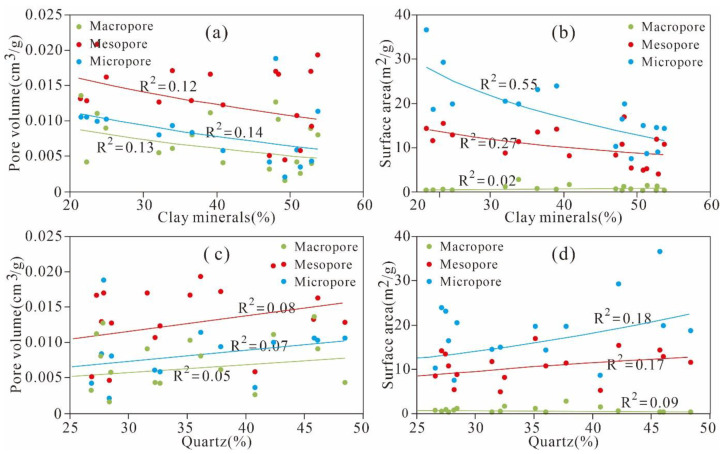
PVs and SAs of different scales versus the clay mineral content (**a**,**b**) and quartz content (**c**,**d**).

As shown in Figure 11a,b, a relatively weak negative correlation is observed between the clay minerals and PV of the micropores, mesopores, and macropores (R^2^ = 0.14, 0.12 and 0.13, respectively). The SA of the micropores and mesopores is negatively correlated with the clay minerals (R^2^ = 0.55, 0.27), whereas the macro-PV exhibits no correlation with the clay minerals. The negative correlations between the clay mineral content and the PV and SA may be caused by the decrease in pore space that occurs because various pores are filled as the clay mineral content increases.

**Figure 12:**
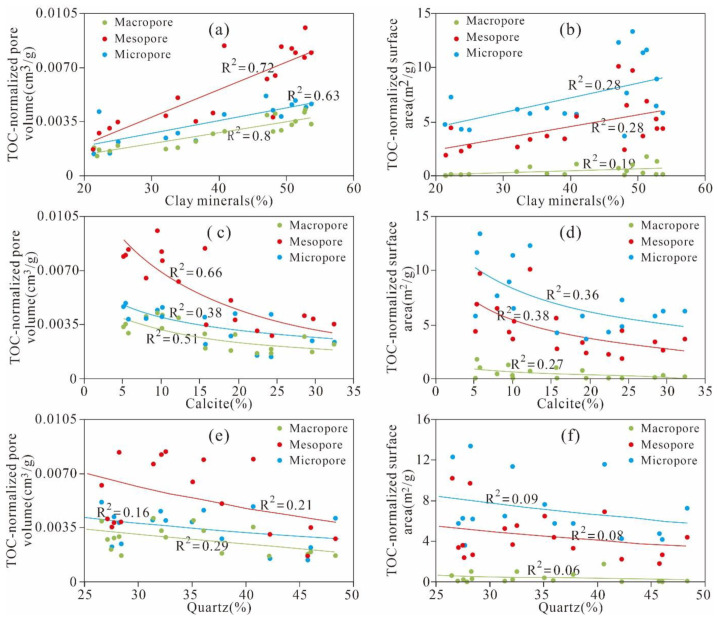
TOC-normalized PVs and SAs of different scales versus the (**a**,**b**) clay mineral content, (**c**,**d**) calcite content, and (**e**,**f**) quartz content.

**Figure 13:**
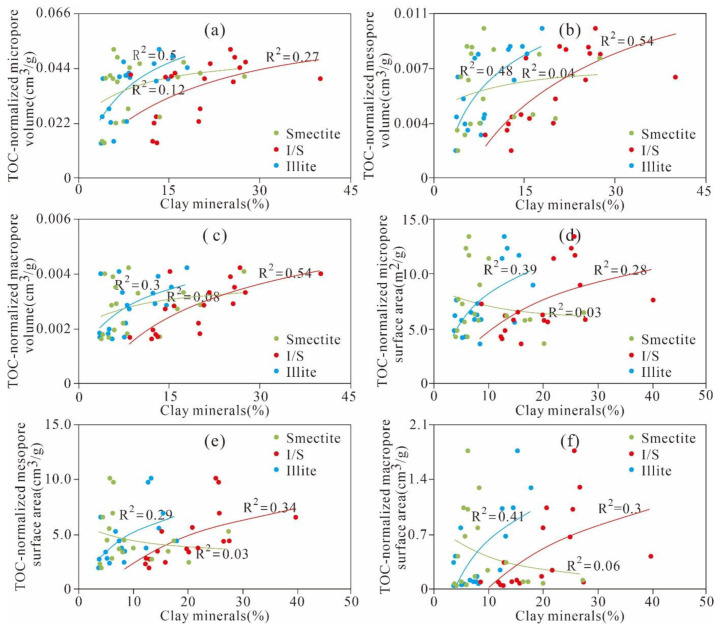
Different clay mineral compositions versus the TOC-normalized PV of the (**a**) micropores, (**b**) mesopores, (**c**) macropores and the TOC-normalized SA of the (**d**) micropores, (**e**) mesopores, (**f**) macropores.

In Figure 11c,d, we observe that almost all of the relationships between the quartz and the PV of the micropores, mesopores, and macropores are relatively weakly positive (R^2^ = 0.07, 0.08 and 0.05, respectively). The relationships between the quartz and the SA of micropores and mesopores are weakly positive (R^2^ = 0.18, 0.17, respectively). The SA of the macropores remains stable as the quartz content changes. The positive relationships between the quartz and the SA and PV of the nanoscale pores are similar to those of the Wufeng–Longmaxi shale from the Jiashiba area, Sichuan Basin of China and the Devonian gas shales in the Horn River Basin of Canada [15,24,38]. However, Yang et al. [42] reported that the Lower Permian Shanxi shale in the Ordos Basin exhibits the opposite tendency. The different relationships between quartz and the pore structure parameters of the PV and SA in different study areas may be due to the diagenetic or sedimentary environment and the origin of quartz [43]. According to the aforementioned discussions, we know that the TOC content is the main factor controlling nanoscale pore structures. To thoroughly analyse the influence of clay minerals and quartz on the nanoscale pore structure, the PV and SA are normalized by the TOC content to remove the obscure TOC content influencing the relationship between the pore structure and clay minerals, calcites, and quartz content. The analysis results show that the clay mineral content exhibits strong positive relationships with the TOC-normalized PVs of the micropores, mesopores, and macropores (R^2^ = 0.63, 0.72 and 0.8, respectively) (Figure 12a). The TOC-normalized PVs of the micropores, mesopores, and macropores exhibit weak positive relationships with the smectite (R^2^ = 0.12, 0.04 and 0.08, respectively) and I/S contents (R^2^ are 0.27, 0.54 and 0.54, respectively) and a relatively strong positive correlation with illite (R^2^ = 0.5, 0.48 and 0.3, respectively) (Figure 13a–c).

The clay minerals are relatively weakly positively correlated with the TOC-normalized SA of micro- and mesopores (R^2^ = 0.28 and 0.28, respectively), whereas they exhibit no obvious relationship with the TOC-normalized SA of macropores (Figure 12b). Further analysis reveals that the TOC-normalized SA of the micropores, mesopores, and macropores exhibits an obvious positive correlation with smectite (R^2^ = 0.03, 0.03 and 0.06, respectively) and a good correlation with I/S (R^2^ = 0.28, 0.34 and 0.30, respectively) but a weak negative correlation with illite (R^2^ = 0.39, 0.29 and 0.41, respectively) (Figure 13d–f). This relationship indicates that I/S and illite are the major contributors to the PV and SA, whereas smectite makes a negligible contribution to the pore structure. The microporosity of clays in shale is related to the clay crystal size and irregular pores between clay frameworks; these results were confirmed by previous studies [1,15,24,44]. In addition, a positive correlation exists between the clay mineral content and the PV and SA, as shown in Figure 12a,b and Figure 13 but not the negative correlation shown in Figure 11a,b. This finding is attributed to the fact that the TOC content obscures the influences of the clay minerals on the PV and SA of the micropores, mesopores, and macropores. The clay minerals containing smectite and I/S in clay-rich shale with low TOC content make a significant contribution to the pore structure in organic-lean shales.

Furthermore, the TOC-normalized PV and SA of the micropores, mesopores, and macropores are negatively correlated with calcite (Figure 12c,d). However, Yang et al. [45] reported that theoretically, the composition cannot have a negative effect on the PV or SA. In reality, the passively negative correlations between calcite and pores of different diameter scales originated from calcite’s negative relationship with clay (Figure 7c).

Additionally, an extremely weak negative correlation is observed between the quartz content and the TOC-normalized PV and SA of the micropores, mesopores, and macropores (Figure 12e,f). Loucks et al. [7] found that the intragranular pores associated with quartz are less abundant in the siliceous mudstones of the Mississippian Barnett shale and that the mechanical compaction and pore blocking of secondary OM and clay floccus results in the destruction of these pores. Therefore, theoretically, quartz makes a minimal contribution to the pore structures of different diameter scales in shale.

It should be noted that although many scholars have analysed the influence of mineral composition on pore structure, most of the research results have not removed the concealment of TOC content on pore structure, and the correlation between each clay mineral composition and pore structure of different scales is rarely discussed under the removal of TOC concealment [23,29,46]. After removing the concealing effect of TOC content on the micro-nano pore structure, this study comprehensively and deeply reveals the influence of brittle minerals such as quartz and calcite, clay minerals such as smectite, I/S and illite, on the formation and development of the PV and SA of micropores, mesopores, and macropore. The influence of the PV and SA of macropores on the formation and development of shale pores is carefully discussed. The control mechanism of mineral composition on pore structure formation and development of shale is discussed in detail. These conclusions are of great significance in revealing the formation and development mechanism of micro-nano pores in Marine shale.

#### 5.3.4. Effect of Maturation on Pore Evolution

**Figure 14:**
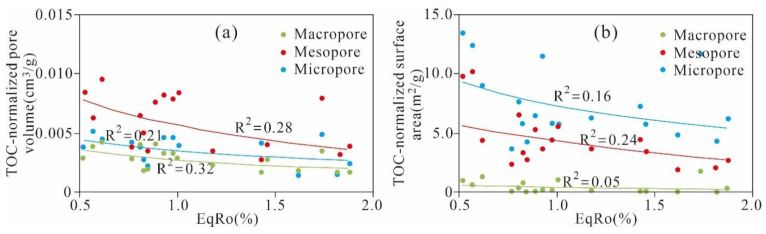
Thermal maturity (EqRo) versus the (**a**) TOC-normalized PV and (**b**) SA of the micropores, mesopores, and macropores.

To obtain information about the effect of thermal maturity on the nanoscale pore structure, this research estimated the relationship between the TOC-normalized PV and SA of different nanoscale pores of Ximaling shale (Figure 14). The results show that the TOC-normalized PV of micropores, mesopores, and macropores are negatively correlated with EqRo (R^2^ = 0.21, 0.28 and 0.32, respectively). The TOC-normalized micropores and mesopore SAs have a relatively weak negative relationship with EqRo (R^2^ = 0.16 and 0.24, respectively), whereas EqRo has a weaker negative effect on the TOC-normalized macropore SA (R^2^ = 0.05). The thermal maturity reflects the dual effects of compaction and time experienced by shale. According to Liu et al. [29], the Xiamaling shale in Zhangjiakou, Hebei has been in a slow subsidence and deep burying state for a long time since its deposition to the end of the Carboniferous; then, this shale started lifting at the beginning of the Permian and subsided rapidly in the middle of the Cretaceous. The negative relationship between EqRo and the TOC-normalized PV and SA of different nanoscale pores of the Ximaling shale is predominantly due to the deep progressive burial and compaction of the shale.

**Figure 15:**
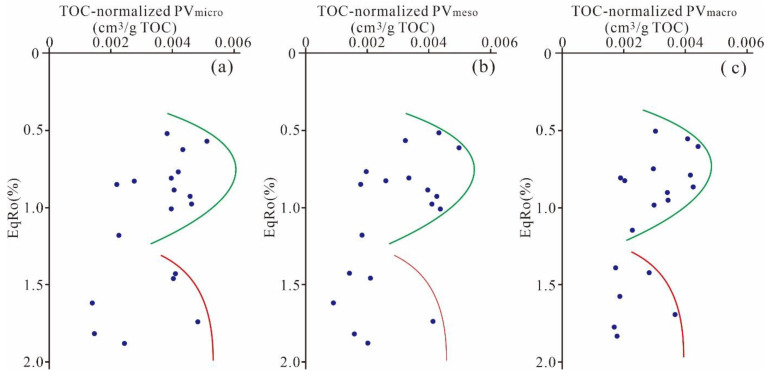
Evolution of TOC-normalized (**a**) micropore, (**b**) mesopore, and (**c**) macropore volume with the equivalent vitrinite reflectance.

Various studies have reported on nanoscale pore evolution with progressing thermal maturity in outcrop shale samples [19,34,47,48,49]. Topór et al. [47] proposed a pore evolution model based on the Silurian Baltic Basin shales with maturation sequences varying from 0.4% to 3.5%. However, the evolution model is ambiguous for Ro > 2.0% given the lack of research dates. Pommer and Milliken [48] established a pore evolution pattern for the Eagle Ford shales, with Ro values ranging between 0.5 and 1.3% but did not interpret the evolution characteristics for shales with maturity greater than 2.0%. To interpret the pore evolution trend with thermal maturity, the TOC-normalized PVs of different diameter scales with progressing EqRo are established based on the Mesoproterozoic Xiamaling shale in Zhangjiakou, Hebei (Figure 15).The increases in the TOC-normalized PV of different diameter scales from the immature to early stage (0.4% < EqRo < 0.7%) mainly result from the progressing burial and compaction, which results in the adjustment of the pore size proportions, especially the conversion of macropores to mesopores and micropores [4,23,50]. Furthermore, the liquid hydrocarbon expulsion of OM and kerogen at a maturity of approximately 0.4–0.7% could also result in an increase in the PV owing to the appearance of organic bubble pores [22]. Additionally, the earlier dissolution and transformation of unstable minerals can contribute to the PV increases at different diameter scales [50,51].

The clear decrease in the TOC-normalized PV of different diameter scales in the middle mature stage with EqRo in the range of 0.7∼1.2% resulted from the oil and bitumen infill in pore spaces, which is also confirmed by the significant increase in the meso- and macro-PVs in the “oil window” after extraction reported by Guo et al. [52]. The obvious decreasing tendency of the TOC-normalized PV of different diameter scales may also result from the adjustment of clay aggregates and continuous compaction.

During the late mature stage (1.2% < EqRo < 2.0%), the significant increase in the TOC-normalized PV of different diameter scales is mainly caused by the secondary cracking of oil and bitumen, accompanied by wet gas generation, which increases the pore space. Hu et al. [53] observed that the porosity increased after Ro 0.9% and reached the maximum value at 1.5∼2.0%. Kuila et al. [54] proposed a significant increase in the PV_<5nm_ for Baltic basin shale with maturity in the range of 1.2∼2.0%. Various similar conclusions can also be found in previous studies [24,34,47,55].

This study found a negative correlation between maturity and shale pore structure, and further analysis found that the TOC-normalized pore volume of micropores, mesopores, and macropores of the shale does not exhibit monotonous increase or decrease. These pores increased first, and then increased again after decrease as the maturity increased to 2.0%. Thus, there is a larger value and a minimum value at 0.7% and 1.2%, respectively. The research conclusion of the influence factors of maturity on pore structure in this study complements the previous research results of shale in high and over matured area, and especially has general applicability to other immature to highly matured marine shale formations of different ages.

### 5.4. Effect of Residual Hydrocarbon on the Pore Structure

#### 5.4.1. Adsorption Desorption Curve Characteristics

**Figure 16:**
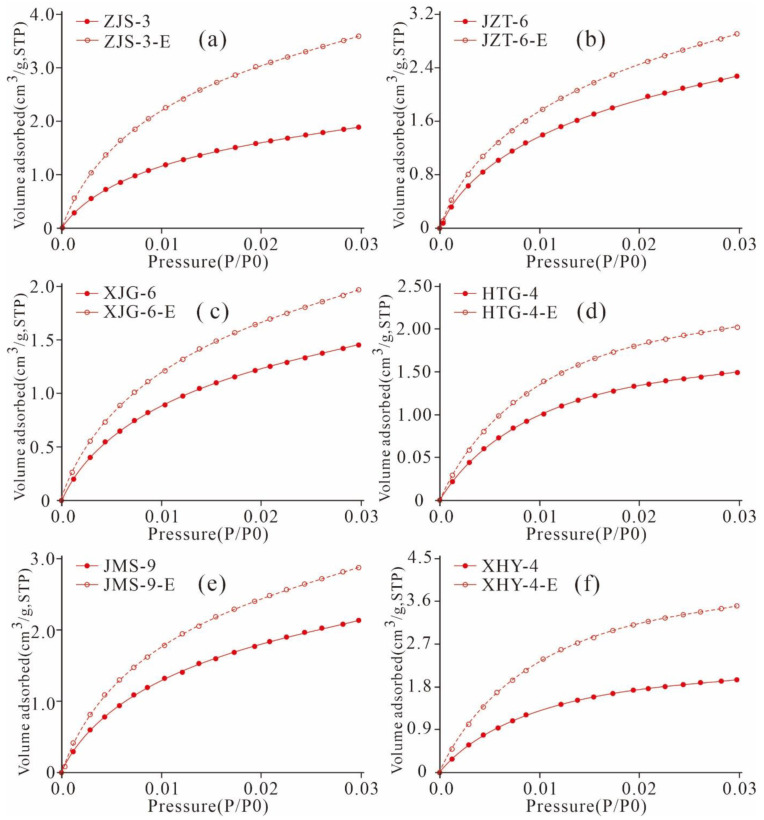
CO_2_ adsorption isotherms of the original shale and extracted shale ((**a**), ZJS-3; (**b**), JZT-6; (**c**), XJG-6; (**d**), HTG-4; (**e**), JMS-9; (**f**), XHY-4).

**Figure 17:**
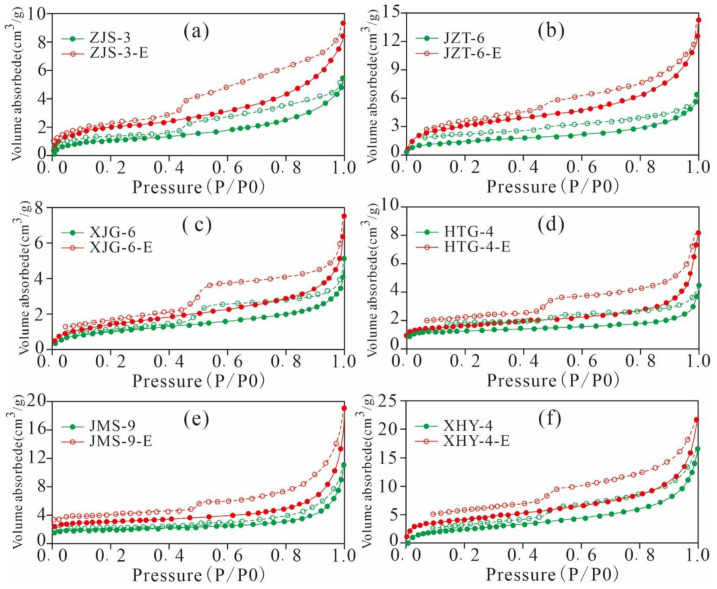
N_2_ adsorption and desorption isotherms of the original shale and extracted shale ((**a**), ZJS-3; (**b**), JZT-6; (**c**), XJG-6; (**d**), HTG-4; (**e**), JMS-9; (**f**), XHY-4).

In this research, low-pressure CO_2_ and N_2_ adsorption were conducted on six original Xiamaling shales and the samples extracted using a 25:2 vol/vol mixture of dicholoromethane and methanol (Figure 16 and Figure 17). The detected samples are of similar OM maturity, type, and mineral composition and content but with different TOC. The result show that CO_2_ and N_2_ adsorption contents of the extracted shales are greater than those of the original shale, indicating that the residual hydrocarbon fills in the pores or blocks the pore throats. When the residual hydrocarbon in the samples is extracted, the pore space is released, and the gas adsorption capacity of the sample increases. Valenza II et al. [56] researched the pore characteristics of shale in the oil generation stage and found that the SA and PV of the extracted samples increased significantly. Jarvia et al. [20] also found that residual bitumen blocked the pores and throats of shale and restricted the adsorption and migration of shale gas. With increasing maturity, the residual bitumen gradually became solid, and its influence on the nanoscale pore structure decreased. Various scholars have found that residual hydrocarbon can block the pores of shale, resulting in a decrease in the SA and PV, supporting our research results [4,34,57].

**Figure 18:**
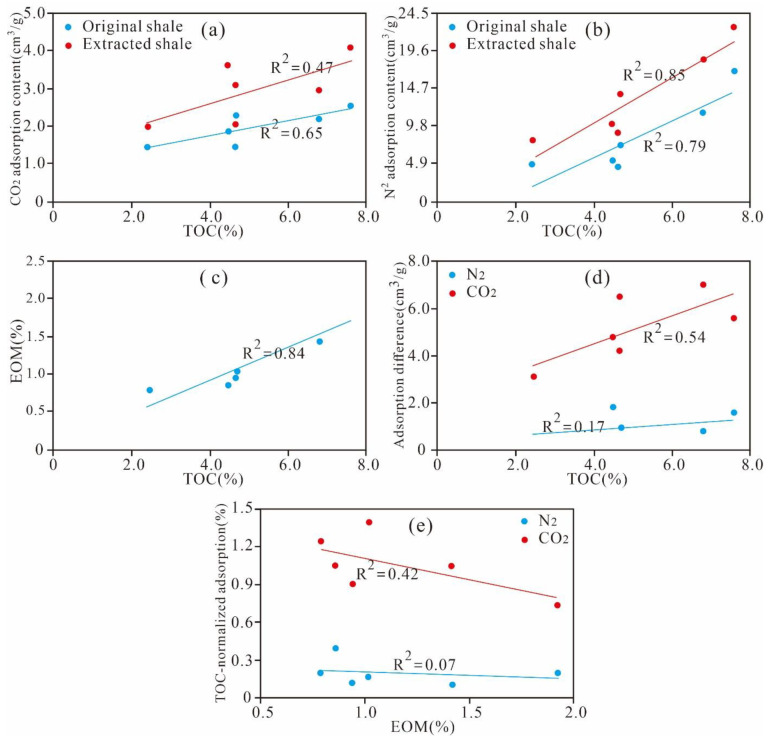
Correlations between gas adsorption content and (**a**,**b**,**d**) TOC content and (**c**,**e**) EOM.

As shown in Figure 18a,b we found that the TOC content is positively correlated with CO_2_/N_2_ adsorption content. The higher the content of TOC, the more micropores and mesopores are enriched in OM at the low maturity stage. In addition, a positive correlation exists between the TOC content and residual hydrocarbon content (R^2^ = 0.84, Figure 18c), and the difference between the adsorption content of the extracted sample and original shale is positively correlated with the TOC content (R^2^ = 0.54 of CO_2_ and 0.17 of N_2_, Figure 18d). These results imply that under the same conditions for OM type, maturity, and mineral components, a higher TOC content indicates a larger amount of residual hydrocarbon generated by shale, with a greater occupied pore space and greater adsorbing capacity after extraction. Furthermore, the negative correlation between the residual hydrocarbon and TOC-normalized adsorbing capacity of CO_2_ and N_2_ (R^2^ = 0.42, 0.07, Figure 18d,e) indicate that a higher content of residual hydrocarbon means a larger extent of blocking of the nanoscale pores of shale. These research results have already been presented in various studies [4,57].

As listed in Table 4, gap values of the micro- and meso-PVs of the extracted shale and original sample are in the ranges of 0.001–0.011 cm^3^/g and 0.001–0.006 cm^3^/g, respectively. Gap values of the micropore and mesopore SAs are in the ranges of 2.309–4.676 m^2^/g and 1.579–4.992 m^2^/g, respectively. The results imply that the pore structures of micropores and mesopores in the extracted samples are significantly larger than those of the original samples. These results indicate that the presence of residual hydrocarbon would occupy the pore space of micropores and mesopores and that the extraction could increase the PV and SA of micropore and mesopores, dredge the micropores and mesopores in shale, and improve the storage capacity and transfusion of shale gas.

#### 5.4.2. Variation Characteristics of the Micropore Structures

**Figure 19:**
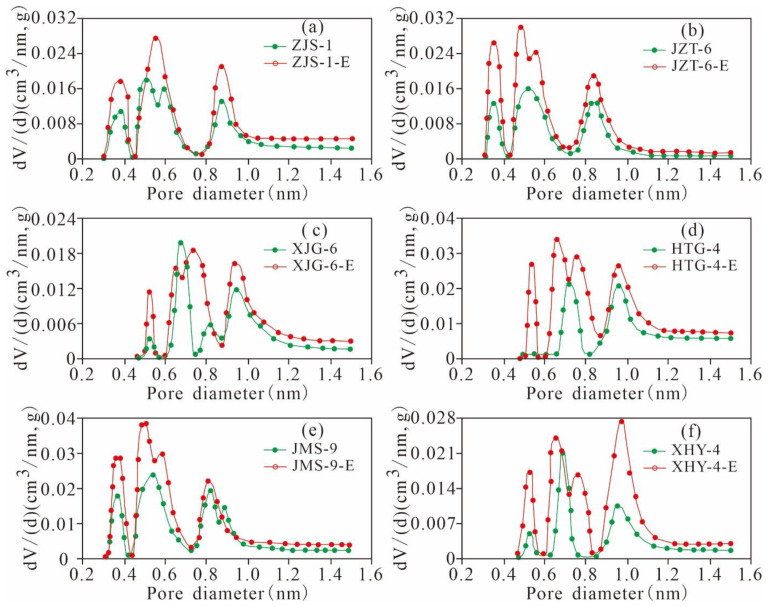
Micro-PV of the original shale and its corresponding extracted sample varies with pore size ((**a**), ZJS-3; (**b**), JZT-6; (**c**), XJG-6; (**d**), HTG-4; (**e**), JMS-9; (**f**), XHY-4).

According to the variation characteristics of the micro-PVs in Table 2 and Figure 19, the micro-PVs of the various pore ranges of the extracted samples increase, and the three-peak distribution characteristics of the PV are constant. In addition, the pores with widths of 0.3~0.4 nm, 0.45–0.6 nm, and 0.7~1.0 nm are the main contributors to the micro-PV, and the rates of change in the PVs of the 3 main peaks are relatively high. These results indicate that the pores with widths of 0.3–1.0 nm exhibit a relatively large PV and provide most of the PV of the micropores, whereas the rate of change in the PV of the pores with widths greater than 1.0 nm is 0, indicating that the micropores with diameters larger than 1 nm make almost no contribution to the PV.

**Figure 20:**
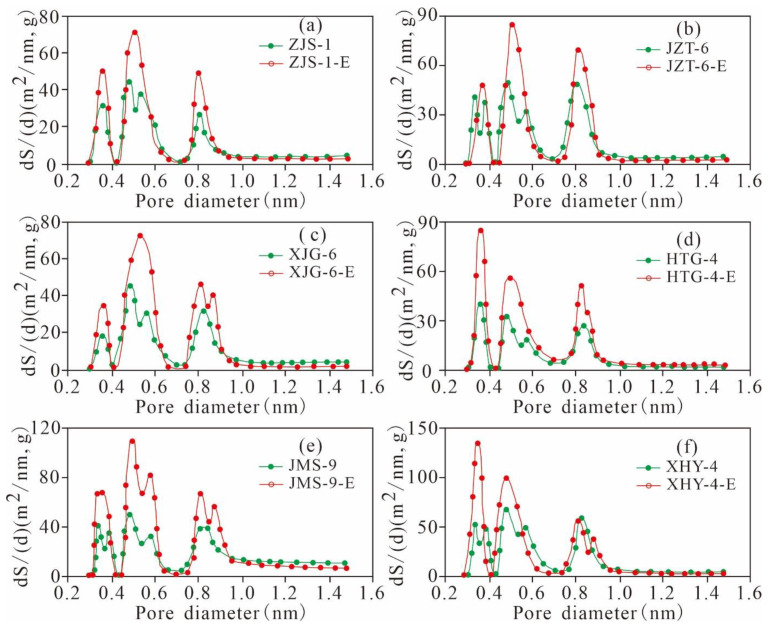
Micropore SA of the original shale and its corresponding extracted sample varies with pore size ((**a**), ZJS-3; (**b**), JZT-6; (**c**), XJG-6; (**d**), HTG-4; (**e**), JMS-9; (**f**), XHY-4).

The SA of the micropores of the six original shales and the extracted samples are distributed in a three-peak manner, and the main peak values are situated at approximately 0.3–0.4 nm, 0.45–0.6 nm, and 0.7–1.0 nm (Figure 20). As the pore diameter increases gradually from 0.3 nm to approximately 1.0 nm, the micropore SA experiences a relatively high rate of change, indicating that the pores of the three peaks have a relatively large SA, contributing most of the SA of the micropores. The rate of change in the SA of the pores with widths greater than 1 nm in various samples is near 0, indicating that the micropores with diameters larger than 1 nm contribute minimally to the SA.

**Figure 21:**
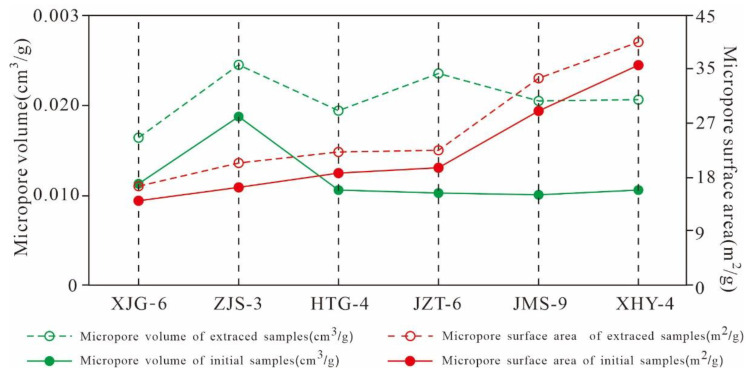
Micropore SA and PV variation characteristics of the original shale and its corresponding extracted samples.

The micropore SA and PV of the 6 extracted shales are greater than those of the original samples. The six samples have different TOC contents and residual hydrocarbon contents, leading to large changes in the micropore SA and PV (Figure 21), indicating that the contents of TOC and residual hydrocarbon greatly influence the micropore structure of shale. The micropore SA and PV of the six samples have similar variation characteristics to that of the pore diameter distribution. The micropore SA and PV of the original shale and the corresponding extracted samples have the same distribution characteristics. The micropore SA and PV are larger at 0.3–1.0 nm, and there is a maximum peak value in the range of 0.45–0.6 nm. However, these parameters are relatively smaller for pore diameters greater than 1 nm and less than 0.3 nm. This finding indicates that the residual hydrocarbon mainly fills micropores with widths of 0.3–1.0 nm, resulting in a significant difference between the micropore SAs and PVs for the original shale and the extracted samples with widths of 0.3–1.0 nm.

**Figure 22:**
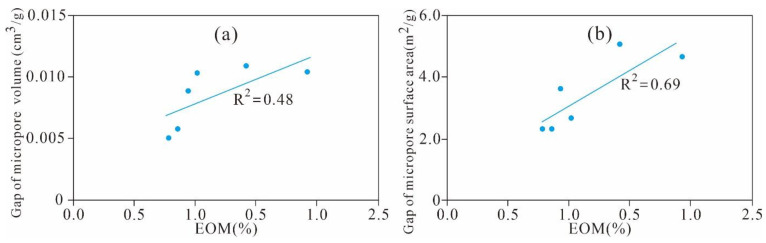
Relationships between the EOM and gap value of the (**a**) micropore volume and (**b**) surface area.

As shown in Figure 22, the extractable OM (EOM) is significantly positively correlated with the gap value of the micro-PV (R^2^ = 0.48) and SA (R^2^ = 0.69). This finding indicates that residual hydrocarbon is one of the main controlling factors for the formation and development of the micropore PV and SA of Xiamaling shale; a higher residual hydrocarbon content occupies a larger micropore SA and PV and results in a larger increase in the micropore SA and PV in the extracted samples.

#### 5.4.3. Variation Characteristics of the Mesopore and Macropore Structures

**Figure 23:**
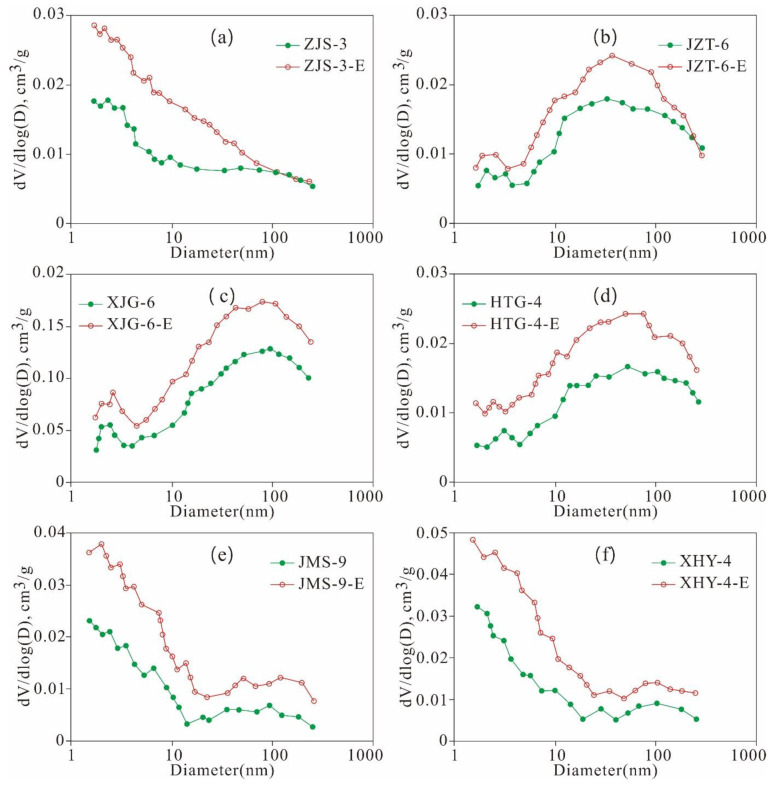
Meso- and macro-PV of the original shale and its corresponding extracted sample varies with the pore size ((**a**), ZJS-3; (**b**), JZT-6; (**c**), XJG-6; (**d**), HTG-4; (**e**), JMS-9; (**f**), XHY-4).

As shown in Figure 23, the meso- and macro-PV of the extracted samples are significantly greater than those of the original shales. ZJS-3, JMS-9, XHY-4 and their extracted samples are of the pre-peak type (Figure 23a,e,f). The pores with smaller diameters contribute relatively more to the PV, and the pores with diameters less than 10 nm contribute the most to the PV. The micropores and mesopores with small diameters constitute the majority of the PV. The PV distributions of JZT-6, XJG-6, and HTG-4 are bi-peak with 2 maximum values at 2 nm and 40 nm and are dominated by the latter (Figure 23b–d).

**Figure 24:**
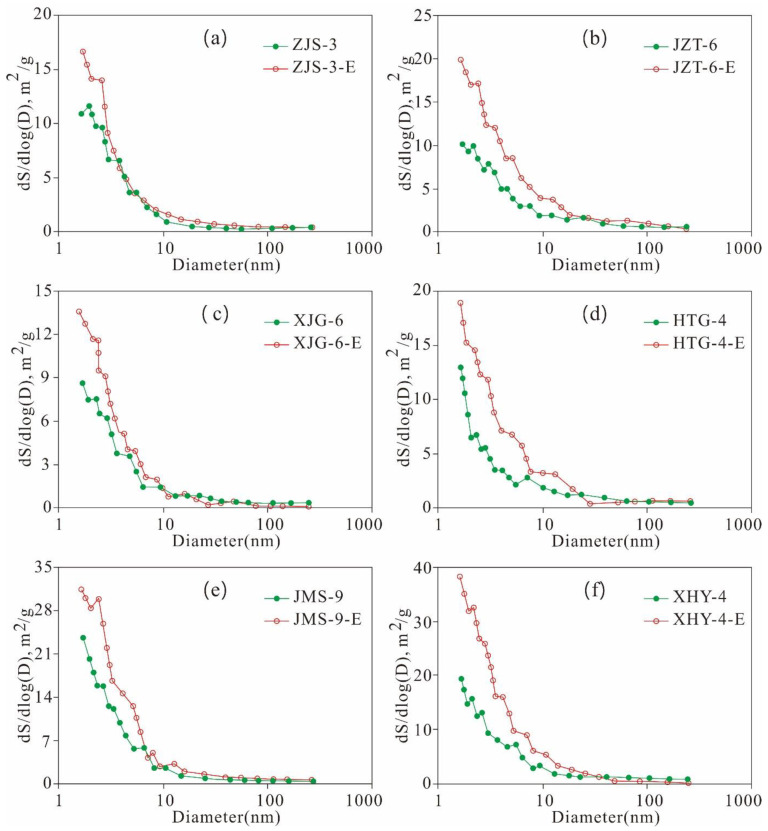
Mesopore and macropore SA of the original shale and its corresponding extracted sample varies with the pore size ((**a**), ZJS-3; (**b**), JZT-6; (**c**), XJG-6; (**d**), HTG-4; (**e**), JMS-9; (**f**), XHY-4).

The SA of the six extracted samples is greater than that of the original shale. The SA of the mesopores and macropores exhibits a unimodal distribution, and the pores with smaller pore diameters contribute more to the SA. Pores with widths less than 10 nm contribute the most to the SA (Figure 24). The SA variation characteristics of the mesopores and macropores are similar to that of the PVs, with some differences, dominated by the following three aspects.

First, the PV distribution of the mesopores and macropores exhibits pre-peak and bi-peak characteristics, whereas the SA distribution shows only pre-peak characteristics. The PV of the pre-peak pores is mainly contributed by pores with widths less than 10 nm, whereas that of the bi-peak pores is mainly contributed by pores with diameters of 2 nm and 40 nm. The SA of the mesopores is mainly contributed by pores with widths less than 10 nm. Second, the mesopore structure variation characteristics of the original shale are similar to those of the extracted samples, and the SAs of the extracted samples are larger than those of the original shale. This finding is attributed to the fact that the pore space is released and increased due to the extraction of the solid residual hydrocarbon in shale, such as solid asphalt. Third, residual hydrocarbon may gather in pores with widths less than 10 nm, as well as those sized 2 nm and 40 nm, so there are significant changes between the original shale and extracted samples regarding the PV and SA of pores with diameters of 2 nm and 40 nm.

**Figure 25:**
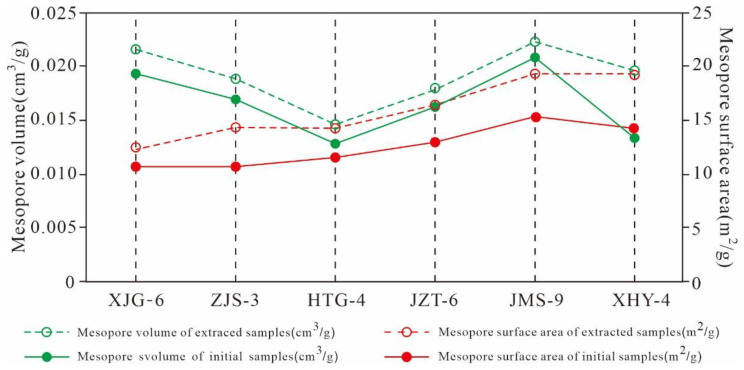
Mesopore SA and PV variation characteristics of the original shale and its corresponding extracted samples.

As shown in Figure 25, the mesopore SA and PV of the six extracted samples are greater than that of the original shale when they were extracted with Soxhlet extraction. There are great differences between the changes in mesopore SA and PV given the different contents of TOC and residual hydrocarbon in the six samples, indicating that the mesopore structure of the shale is also controlled by the TOC and residual hydrocarbon contents. The mesopore SA and PV distribution characteristics of the six original shale samples are the same as those of the extracted samples, and pores with diameters less than 10 nm, 2 nm, and 40 nm may be where residual hydrocarbon is stored. These characteristics result in significant difference between the SA and PV of the pre-peak type mesopores with widths less than 10 nm of the original shale and those of the extracted samples, as well as that of the PV of the bi-peak type mesopores with widths of 2 nm and 40 nm.

**Figure 26:**
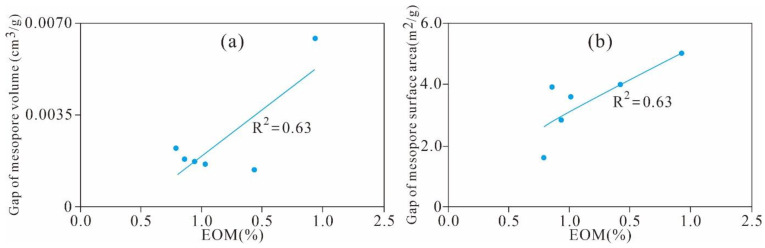
Relationships between the EOM and gap value of the (**a**) mesopore volume and (**b**) surface area.

The EOM and gap value of meso-PV and SA exhibits an obvious positive correlation with coefficients of 0.63 and 0.63, respectively (Figure 26a,b). This finding implied that residual hydrocarbon have a significant effect on the formation and development of the mesopore SA and PV of Xiamaling shale. The higher the content of residual hydrocarbon, the larger the mesopore SA and PV it occupies, and the larger the SA and PV of the mesopore of the sample after extraction, which is consistent with the correlation between residual hydrocarbon and micropore structure.

It is believed that residual hydrocarbons can block pores and throats of shale, and have a certain restrictive effect on gas adsorption and migration. However, few scholars have reported that residual hydrocarbons specifically affect the pore of that pore size, and reports about the correlation between residual hydrocarbon content and SA and PV of different scales are scarce [4,20,34,56,58,59]. This study clearly shows that residual hydrocarbons mainly affect the changes in the pore structure parameters, reveals the degree of restriction on the SA and PV of pores with different diameter, clarifies positive correlation between residual hydrocarbon content and SA and PV of pores with different scales, and reveals the main storage space of residual hydrocarbon in the reservoir and its influence on reservoir porosity characterization. Residual hydrocarbons have an important impact on the gas-bearing properties of matured shale. These studies provide important evidence for understanding and evaluating the adsorption, desorption, diffusion, and migration of shale gas.

## 6. Conclusions

(1) MIP curve of the Xiamaling shale can be divided into three types, which represent three different sets of pore characteristics and reflect different pore connectivity performances. The N_2_ isotherm curve can be roughly classified into four types, denoting four different types of mesopores. The N_2_ PV distribution exhibits a pre-peaks, bi-peaks, and post-peaks due to the influences of the TOC content and mineral components, whereas the micropore SA and PV exhibits a three-peak type distribution. The full-scale distribution characteristics of the samples are multimodal with multiple scales, bi-peaks with primarily mesopores or micropores.

(2) OM contributes mostly to the development of micro- and mesopores. Clay minerals, mainly smectite and I/S, are the main contributors to the TOC-normalized PV and SA of the micropores, mesopores, and macropores. Illite is positively correlated with the TOC-normalized PV, but is negatively correlated with the TOC-normalized SA of pores with different diameter scales.

(3) The thermal maturity controls the generation and development of pores at different diameter scales. Additionally, this research establishes a general TOC-normalized PV evolution mode.

(4) The micropore and mesopore parameters are significant affected by TOC and residual hydrocarbon content. A increased residual hydrocarbon content indicates a more occupied pore space. The residual hydrocarbon mainly fills micropores with diameters of 0.3–1.0 nm and mesopores with widths less than 10 nm and those of 2 nm and 40 nm. The SA and PV of the extracted sample increase significantly due to the release of the occupied pore space.

## Figures and Tables

**Table 1:** Geochemical information and mineralogical composition for the investigated Xiamaling shales.

Sample ID	Formation	TOC (%)	EqRo (%)	Mineralogical Composition Relative Percent (%)									EOM (%)
				Quartz	Calcite	Feldspar	Pyrite	Clays	Smectite	I/S	Illite	Chlorite	
ZJS-1	Q_nx_	2.23	0.89	31.5	10.2	3.1	2.4	52.8	6.7	15.2	27.4	3.5	–
ZJS-3	Q_nx_	4.48	0.77	27.8	19.6	2.3	2.2	48.1	8.2	15.8	20.1	4.0	0.86
ZJS-6	Q_nx_	2.58	0.81	35.2	8.1	4.7	3.6	48.4	4.0	40.0	4.4	0	–
JZT-1	Q_nx_	3.41	0.83	37.8	19.1	4.0	5.1	34.0	4.9	20.1	8.1	0.9	–
JZT-6	Q_nx_	4.69	0.85	46.1	15.8	8.2	4.9	25.0	5.1	12.3	6.0	1.6	1.02
XJG-3	Q_nx_	1.31	0.93	32.2	10.1	4.9	1.9	50.9	12.3	21.8	9.9	6.9	–
XJG-6	Q_nx_	2.45	0.98	36.1	5.2	3.5	1.4	53.8	7.3	27.6	17.4	1.5	0.79
XJG-9	Q_nx_	1.47	1.01	32.6	15.7	6.7	4.1	40.9	14.6	20.8	5.5	0	–
HTG-2	Q_nx_	4.12	1.46	27.2	28.6	3.7	1.3	39.2	8.4	14.4	16.4	0	–
HTG-3	Q_nx_	3.67	1.18	27.6	32.4	1.7	1.7	36.6	7.7	19.8	7.5	1.6	–
HTG-4	Q_nx_	4.66	1.43	48.4	24.3	1.9	3.1	22.3	7.8	8.5	5.1	0.9	0.94
HTG-5	Q_nx_	3.28	1.88	28.5	29.6	8.6	1.1	32.2	3.7	13.1	13.3	2.1	–
JMS-4	Q_nx_	0.55	0.52	28.3	5.8	9.4	7.2	49.3	12.7	25.6	6.3	4.7	–
JMS-5	Q_nx_	0.97	0.62	21.8	9.6	11.3	4.4	52.9	17.9	26.7	8.3	0	–
JMS-9	Q_nx_	6.80	1.82	42.3	22.5	11.4	0	23.8	5.6	12.1	3.8	2.3	1.42
XHY-3	Q_nx_	0.82	0.57	26.7	12.3	10.7	3.1	47.2	13.2	25.1	5.7	3.2	–
XHY-4	Q_nx_	7.60	1.62	45.8	24.3	5.1	3.3	21.5	3.6	12.8	4.1	1.0	1.93
XHY-7	Q_nx_	0.74	1.74	40.7	5.4	2.5	0	51.4	15.4	25.8	6.2	4.0	–

Note: “EqRo” indicates equivalent vitrinite reflectance; “–” indicates no data; “I/S” indicates illite-smectite mixed layer; “EOM” indicates extracted OM content.

**Table 2:** MIP measurement results of the Mesoproterozoic Xiamaling shale in Zhangjiakou, Hebei.

Sample ID	Macropore PV (cm^3^/g)	Macropore SA (m^2^/g)	Bulk Density (g/cm^3^)	Skeletal Density (g/cm^3^)	Porosity (%)
ZJS-1	0.0091	0.2414	2.2201	2.2614	1.84
ZJS-3	0.0127	0.2909	2.1961	2.2345	1.72
ZJS-6	0.0103	1.0617	2.3139	2.3456	1.35
JZT-1	0.0062	2.6703	2.2421	2.2644	0.99
JZT-6	0.0091	0.2122	2.2802	2.3332	2.27
XJG-3	0.0043	0.3128	2.2146	2.2345	0.89
XJG-6	0.0081	0.2139	2.3062	2.3511	1.91
XJG-9	0.0042	1.5341	2.1791	2.2163	1.68
HTG-2	0.0112	0.3629	2.3577	2.3712	0.57
HTG-3	0.0081	0.5828	2.2523	2.2654	0.58
HTG-4	0.0043	0.2227	2.1987	2.2625	2.82
HTG-5	0.0056	1.0839	2.3513	2.3743	0.97
JMS-4	0.0016	0.5642	2.3389	2.3683	1.24
JMS-5	0.0041	1.2618	2.3581	2.3825	1.03
JMS-9	0.0111	0.3628	2.3079	2.3847	3.22
XHY-3	0.0032	0.5509	2.6341	2.6641	1.13
XHY-4	0.0136	0.2642	2.2755	2.3454	2.98
XHY-7	0.0026	1.3133	2.3903	2.4314	1.69

**Table 3:** Main pore structure parameters measured by low-pressure gas adsorption of the Mesoproterozoic Xiamaling shale in Zhangjiakou, Hebei.

Sample ID	N_2_ Adsorption			CO_2_ Adsorption		
	V_BJH_ (cm^3^/g)	S_BET_ (m^2^/g)	APD (nm)	V_DFT_ (cm^3^/g)	S_DFT_ (m^2^/g)	APD (nm)
ZJS-1	0.01701	11.7	22.69	0.00907	14.4	0.885
ZJS-3	0.01699	10.7	12.34	0.01882	16.4	0.845
ZJS-3-E	0.01881	14.6	16.87	0.02449	18.7	1.236
ZJS-6	0.01667	16.9	4.11	0.01026	19.7	0.554
JZT-1	0.01715	11.4	16.23	0.00942	19.7	0.886
JZT-6	0.01626	12.8	11.63	0.01931	19.8	0.995
JZT-6-E	0.01784	16.4	14.32	0.02148	22.5	1.689
XJG-3	0.01075	4.8	10.48	0.00601	14.9	0.596
XJG-6	0.01935	10.7	4.45	0.01137	14.3	0.972
XJG-6-E	0.02154	12.2	6.89	0.01639	16.6	1.554
XJG-9	0.01234	8.1	4.22	0.00584	8.2	1.511
HTG-2	0.01664	14.1	7.84	0.01666	23.8	0.885
HTG-3	0.01293	13.5	9.36	0.00833	23.0	1.287
HTG-4	0.01286	11.4	8.04	0.01063	18.7	0.844
HTG-4-E	0.01456	14.2	12.89	0.01945	22.3	1.248
HTG-5	0.01271	8.8	9.66	0.00803	20.4	1.567
JMS-4	0.00459	5.4	3.98	0.00211	7.4	1.264
JMS-5	0.00925	4.2	5.54	0.00428	8.7	1.563
JMS-9	0.02081	15.4	8.53	0.01002	29.3	1.267
JMS-9-E	0.02221	19.3	13.05	0.02086	34.3	1.994
XHY-3	0.00512	8.3	3.66	0.00422	10.1	0.875
XHY-4	0.01324	14.3	4.09	0.01262	36.6	0.866
XHY-4-E	0.01963	19.3	9.29	0.02093	41.3	1.632
XHY-7	0.00589	5.1	3.54	0.00359	8.6	0.894

Note: S_DFT_ = SA using density functional theory; V_DFT_ = PV using density functional theory; S_BET_ = SA using the Brunauer-Emmett-Teller method; V_BJH_ = PV using the Barrett-Joyner-Halenda method; APD = average pore diameter.

**Table 4:** Gap values between the micropore and mesopore PVs and SAs in the extracted samples and original shales.

Sample ID	CO _2_ Adsorption		N_2_ Adsorption	
	Gap of V_DFT_ (cm^3^/g)	Gap of S_DFT_ (m^2^/g)	Gap of V_BJH_ (cm^3^/g)	Gap of S_BET_ (m^2^/g)
ZJS-3	0.006	2.309	0.002	3.890
JZT-6	0.010	2.620	0.002	3.551
XJG-6	0.005	2.311	0.002	1.579
HTG-4	0.009	3.625	0.002	2.807
JMS-9	0.011	5.060	0.001	3.967
XHY-4	0.001	4.676	0.006	4.992

## Data Availability

Not applicable.
